# Ultrasonic predator–prey interactions in water–convergent evolution with insects and bats in air?

**DOI:** 10.3389/fphys.2013.00137

**Published:** 2013-06-12

**Authors:** Maria Wilson, Magnus Wahlberg, Annemarie Surlykke, Peter Teglberg Madsen

**Affiliations:** ^1^Department of Bioscience, The Faculty of Mathematics and Natural Sciences, University of OsloOslo, Norway; ^2^Institute of Biology, University of Southern DenmarkOdense, Denmark; ^3^Zoophysiology, Department of Bioscience, Aarhus UniversityAarhus, Denmark

**Keywords:** predator–prey interaction, echolocation, ultrasound, toothed whale, Alosinae, bat, moth, evasivemaneuvers

## Abstract

Toothed whales and bats have independently evolved biosonar systems to navigate and locate and catch prey. Such active sensing allows them to operate in darkness, but with the potential cost of warning prey by the emission of intense ultrasonic signals. At least six orders of nocturnal insects have independently evolved ears sensitive to ultrasound and exhibit evasive maneuvers when exposed to bat calls. Among aquatic prey on the other hand, the ability to detect and avoid ultrasound emitting predators seems to be limited to only one subfamily of Clupeidae: the Alosinae (shad and menhaden). These differences are likely rooted in the different physical properties of air and water where cuticular mechanoreceptors have been adapted to serve as ultrasound sensitive ears, whereas ultrasound detection in water have called for sensory cells mechanically connected to highly specialized gas volumes that can oscillate at high frequencies. In addition, there are most likely differences in the risk of predation between insects and fish from echolocating predators. The selection pressure among insects for evolving ultrasound sensitive ears is high, because essentially all nocturnal predation on flying insects stems from echolocating bats. In the interaction between toothed whales and their prey the selection pressure seems weaker, because toothed whales are by no means the only marine predators placing a selection pressure on their prey to evolve specific means to detect and avoid them. Toothed whales can generate extremely intense sound pressure levels, and it has been suggested that they may use these to debilitate prey. Recent experiments, however, show that neither fish with swim bladders, nor squid are debilitated by such signals. This strongly suggests that the production of high amplitude ultrasonic clicks serve the function of improving the detection range of the toothed whale biosonar system rather than debilitation of prey.

## Introduction

Predation is one of the major driving forces in the evolution of the morphology and behavior of organisms (Dawkins and Krebs, 1987; Vermeij, 2002). In tight predator–prey interactions, the natural selection pressure for evolving abilities to detect and catch, or detect and avoid, the other part can be strong and lead to an evolutionary arms race, where adaptations in one species lead to counter adaptations in the other (Dawkins and Krebs, 1987; Dielt and Kelly, 2002).

A classic neuroethological example of how predation and the sensory means of predators have affected the life and sensory systems of prey organisms is the interaction between echolocating bats and ultrasound detecting nocturnal insects, in particular moths. Bats emit intense ultrasonic calls and use the echoes reflected off objects to search for and capture prey (Griffin, 1958; Schnitzler et al., 2003; Schnitzler and Kalko, 2008; Moss and Surlykke, 2010). Bats are important nocturnal predators and therefore place a strong selection pressure on their prey to evolve means to detect and avoid them (Kalka et al., 2008). This selection pressure has driven the evolution of ears sensitive to ultrasonic bat calls in eight moth families (Miller and Surlykke, 2001). Kenneth Roeder, a pioneer in the research of ultrasonic hearing in insects, conducted in the fifties and sixties behavioral experiments where he exposed moths to ultrasonic signals mimicking bat echolocation calls. He found that moths exhibit a complex pattern of anti-predator responses depending on the repetition rate as well as the intensity of the echolocation signals impinging on them (Roeder, 1964, 1967; Miller and Surlykke, 2001). When moths are exposed to low-intensity ultrasonic bat calls, they exhibit negative phonotactic behavior, where they turn and fly directly away from the sound source with increased flying speed. If moths are exposed to high-intensity ultrasonic calls mimicking a bat just before a prey-capture attempt, they will exhibit an erratic evasive response with unpredictable flight patterns that often ends in a power dive or passive drop toward the ground (Fullard, 1998; Fullard et al., 2008; Jacobs et al., 2008). Thus, not only do moths react when exposed to bat calls, they also exhibit an anti-predator response that is correlated with the strength of the predation risk.

It is not only moths that have evolved ears sensitive to ultrasound. Bats feed on a variety of nocturnal insects (Fullard, 1998) and it is generally accepted that the heavy predation pressure from echolocating bats has led to convergent evolution of ears sensitive to ultrasonic signals in at least six orders of insects; Lepidoptera (8 families of nocturnal moths), Neuroptera (lacewings), Coleoptera (beetles), Dictyoptera Mantodea (praying mantids), Orthoptera (katydids, crickets and grasshoppers), and Diptera (parasitic fly species) (Yack and Fullard, 1993; Hoy and Robert, 1996; Yack and Fullard, 2000; Conner and Corcoran, 2012). The ultrasound sensitive ears in combination with sudden evasive maneuvers mitigate predation risk from echolocating bats, increasing the insect's chance of survival by at least 40% (Surlykke et al., 1999). Some bats have lowered the intensity of their calls by 20–40 dB, apparently as a counterstrategy against the ultrasound sensitive ears (Goerlitz et al., 2010). While other bats echolocate at frequencies outside the best hearing range of moths (Fullard, 1998; Fullard et al., 2008; ter Hofstede et al., in press). Both strategies appear to serve the same purpose of rendering the signals difficult to detect by insect prey (Fullard, 1998). Some bats may also broaden their echolocation beam in the last phase of pursuit to keep the insect within their “acoustic field of view” in spite of evasive maneuvers (Jakobsen and Surlykke, 2010). Thus, in the predator–prey interactions of bats and insects there are examples of both strategies and counterstrategies by prey and predator.

Like bats, echolocating toothed whales use a highly advanced biosonar system to detect and catch prey. It has therefore been suggested that despite the very different physical environments of air and water a similar acoustic predator–prey arms race should exist between echolocating toothed whales and their prey (Mann et al., 1998; Astrup, 1999). During the last 15 years several studies have focused on toothed whales and their prey and in the light of the new results we here seek to address and discuss the possible convergent evolution in the acoustic interactions between bat–insect and toothed whale–prey interactions. We do that by providing a brief overview of differences and similarities of echolocation in bats and toothed whales and discuss the implications for biosonar behavior in the two mammalian groups. Then we compare the defense strategies in marine prey with defense strategies in nocturnal insects and discuss the functional basis for developing sensory systems to detect ultrasonic echolocation signals emitted by toothed whales and bats.

## Echolocation in bats and toothed whales

Echolocation is an active sensory process where the echolocating animal emits the sound energy which it subsequently hears as echoes reflected off objects ahead of it. Information is then extracted from the environment by the acoustic features of the returning echo and by the delay from sound emission to echo detection. The approximate echo level (EL) returning to the echolocating animal can be estimated using the active sonar equation that includes the target strength (TS), the source level of the emitted sound pulse (SL) and the transmission loss (TL) (all in dB) (Urick, 1983):

(1) EL=SL+TS−2×TL

Detection of a returning echo is possible when the EL is higher than the hearing threshold of the echolocating animal or higher than the ambient noise or clutter levels if they surpass the hearing threshold. To forage successfully with sound, echolocating animals in both air and water engage in the phases of search, approach and capture of prey as defined by Griffin (1958). However, air and water are physically two very different types of media and therefore offer very different conditions for the production, transmission, and reflection of sound (Madsen and Surlykke, 2013). The sound speed and density in air are considerably lower than in water which results in very different acoustic impedances of the two media.

Bat echolocation calls can reach SLs of up to 140 dB re 20 μPa (pp) at 0.1 m in air (Surlykke and Kalko, 2008), whereas most toothed whales generate SLs up to 225 dB re 1 μPa (pp) at 1 m in water (Au, 1993; Madsen et al., 2004; Wahlberg and Surlykke, 2013). However, source levels should not be compared directly across the water–air interface. First, the source levels in air and water are given with different reference values and different reference distances. Secondly, the acoustic impedance, given by the ratio of the acoustic pressure and particle motion of an acoustic wave, is much lower in air than in water. This makes it more difficult to generate high-intensity acoustic signals in air than in water. Actually, the sound levels emitted by bats are close to the upper limit of efficient sound production in air. Bats apparently compensate for this restriction by emitting pulses that are relatively long, up to 30–1000 times longer than toothed whale echolocation clicks. This means that the bat sound pulses will carry more energy for a given sound pressure level. When we take these different durations of the signals and the different impedances of the medium into account, a 2 ms bat call in air with a SL of 140 dB re 20 μPa (pp) at 0.1 m has an energy flux density of around 5 × 10^−5^ J/m^2^ and a 50 μs long toothed whale click with a source level of 225 dB re 1 μPa (pp) at 1m has an energy flux density of 4 × 10^−2^ J/m^2^. Thus, bat calls in air are emitted with an energy content about 3 orders of magnitude below those of toothed whale clicks (Madsen and Surlykke, 2013). Both signals are, however, among the highest biologically produced sound intensities found in either media.

Another important difference between air and water is that the sound speeds vary by almost a factor of five between the two media. The wavelength at a given frequency will therefore be almost five times longer in water compared to air. Wavelengths are important for biosonar operation in two ways: (1) for the generation of directional sound beams to increase the SL and decrease clutter levels, and (2) to ensure geometric backscatter from targets of interest, and to extract information of the physical properties of the target by detecting interference patterns generated by multiple reflections at different parts of the target. Geometric backscatter for most prey sizes of interest for bats and toothed whales, will occur when their biosonars operate at frequencies higher than 5–15 kHz depending on prey size (Madsen and Surlykke, 2013). However, many species of both bats and toothed whales produce sound for echolocation at much higher frequencies scaled inversely to their body size. To achieve high directionality of the transmitting beam, an echolocating animal must produce sounds at short wavelengths relative to the size of their transmitting aperture. Small animals must hence use higher frequencies to produce the same directionality as larger specimen. The directionality of sound production can be quantified using the directionality index, DI. This is the source level difference (in decibels) between the directional source in question and an omnidirectional source emitting the same power (Au and Hastings, 2008). Bats seem to operate their biosonars with directionality indices between 11 and 18 dB (Jakobsen et al., 2013), while toothed whales operate their sound beams with DIs from 24 to 32 dB (Wahlberg and Surlykke, 2013). The price to pay for using higher frequencies in small echolocating species is that the frequency dependent absorption is high. The effect is much more dramatic in air which is likely explaining why bats operate at lower frequencies compared to their size than toothed whales (Madsen and Surlykke, 2013). Consequently, most bats and toothed whales emit sonar pulses in a similar frequency range from 15 to 150 kHz.

From the above-mentioned source levels of bats and whales the estimated prey detection ranges of bats are 3–10 m (Holderied and Helversen, 2003; Jung et al., 2007) whereas the estimated prey detection ranges of toothed whales are 15–325 m (Au et al., 2007; Madsen et al., 2007). The huge difference in detection ranges between bats and whales is mainly caused by whales using much higher source levels and the sound absorption being much lower in water. It might therefore be expected that toothed whales would produce sonar pulses at slower rates than bats because the two way travel ranges to their prey targets are much longer. However, because of the almost five times faster speed of sound in water compared to in air, toothed whales have two-way travel times that are almost five times shorter than bats for a certain target range. This results in surprisingly similar biosonar sampling rates for most species of bats and toothed whales (Madsen and Surlykke, 2013).

Both bats and toothed whales employ various versions of the Griffin model of search, approach and capture, where the inter-pulse intervals and output levels are reduced with range to the prey (Griffin, 1958; Au and Benoit-Bird, 2003; Jensen et al., 2009). Not all species reduce output levels and ICI's in the approach phase (Madsen et al., 2005), but all studied echolocating bats and toothed whales in the wild employ fast repetition rates in the so-called buzz during prey capture attempts, when hunting for moving prey (DeRuiter et al., 2009; Madsen and Surlykke, 2013; Ratcliffe et al., 2013).

Thus despite the vast differences in size of bats and toothed whales and the very different media in which they operate their biosonars, echolocation necessitates the exposure of prey items to high ultrasonic sound levels at high pulse rates. It follows that these predators loudly announce their presence to prey and predators equipped with sensory means to detect them.

## Big bang—or not?

Toothed whales can generate very intense sound pressure levels up to 225 dB re 1 μPa (pp) (Au, 1993), in the case of the sperm whale even up to 240 dB re 1 μPa (pp) (Møhl et al., 2003); the highest known sound pressure generated by any animal. The reason why toothed whales produce such high sound pressure levels has been lively debated. It clearly enables the animal to detect prey items at longer ranges, or prey items with low target strengths (Equation 1). However, it has also been speculated that the intense ultrasonic clicks not only play a role in echolocation but also helps the whale to catch prey by acoustic debilitation (Berzin, 1971; Norris and Møhl, 1983). Such a dramatic use of sound is known from another aquatic predator–prey interaction between snapping shrimps and their prey. Snapping shrimps make broadband clicks by an extremely rapid closure of the specialized snapper claw, (Herberholz and Schmitz, 1999). The clicks are produced by the collapse of cavitation bubbles generated in a fast flowing water jet during claw closure (Versluis et al., 2000). The clicks can give rise to sound levels of 220 dB re 1 μPa (pp) at close range. A single snap from the claw seems to be sufficient to stun the prey (reviewed by Herberholz and Schmitz, 1999). It is therefore tempting to speculate that toothed whales may use sound in a similar manner. A major difference between snapping shrimps and whales is, however, that the prey of snapping shrimp is exposed to a water jet with particle accelerations much higher than what even a free field pressure of 240 dB re 1 μPa (pp) would predict. It is not known whether it is the sound pressure or particle acceleration that debilitates the prey. Therefore, the fact that snapping shrimps may be able to debilitate prey does not necessarily mean it is possible for the toothed whale to do the same, even though the emitted pressure levels for the toothed whale can be higher than for the snapping shrimp.

Nevertheless, several early experiments did lend support to this so-called biological big bang hypothesis by demonstrating that high exposure levels could disorient fish (Zagaeski, 1987; Mackay and Pegg, 1988; Marten and Norris, 1988). However, many of these experiments used stimuli with very little spectral and temporal resemblance to toothed whale echolocation clicks (Zagaeski, 1987; Mackay and Pegg, 1988; Marten and Norris, 1988). Zagaeski (1987) successfully debilitated guppies with an exposure level of more than 230 dB re 1 μPa (pp), generated with a spark generator. Norris and Møhl (1983) fired small blasting caps in the vicinity of several species of small cephalopods with little evidence of debilitation. In both these experiments the spectral content of the stimuli had a low frequency emphasis and the rise time of the signals was much faster compared to a toothed whale echolocation click. In addition, the source was very close to the animal. The fast rise time of the stimuli and the close proximity between the animal and the source may both induce a large excess particle motion, which can cause damage to the fish tissue that would not be observed using more realistic signals and ranges.

**Figure 1:**
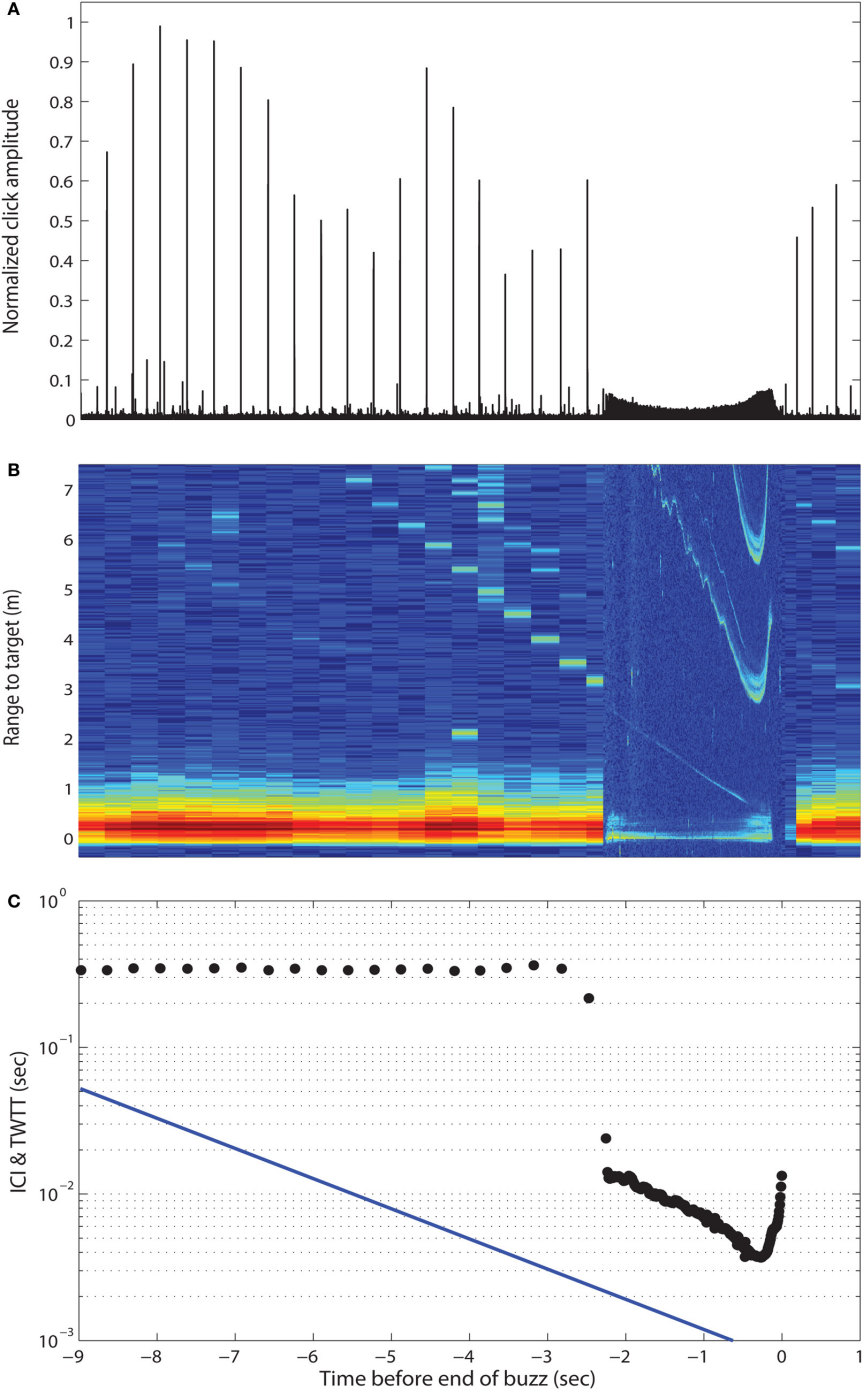
**Approach and buzz phases of an echolocating Blainville's beaked whale. (A)** Envelopes of the emitted clicks as time from end of buzz. Note the dramatic change in click amplitudes during buzzing. **(B)** Echogram of the emitted clicks and echoes from the approached prey. **(C)** Interclick interval (ICI) and two-way travel time (TWTT). Adapted with permission from Madsen et al. (2013).

During the last 15 years, our knowledge of toothed whale echolocation signals has increased along with the capability to reproduce them in the laboratory. Experiments using simulated echolocation signals at ultrasonic frequencies with exposure levels up to 226 dB re 1 μPa (pp) and repetition rates of up to 200 clicks/s, show that neither squid (Wilson et al., 2007) nor fish with swim bladders (Benoit-Bird et al., 2006; Schack et al., 2008) are debilitated by intense ultrasonic pulse trains. The obvious question is whether the exposure levels in these controlled debilitation trials are representative of the levels evoked by echolocating toothed whales in the wild. Deployments of sound recording tags on foraging toothed whales have shed light on that issue and shown that toothed whales consistently reduce their source level 20 dB or more when they initiate the buzz phase about a body length from their prey (Madsen et al., 2002, 2005; DeRuiter et al., 2009) (Figure 1). Therefore, echolocating toothed whales do not maximize the impinging sound pressure level on their prey, as would be expected if they were attempting to debilitate it. In fact, none of the estimated received sound pressure levels prior to or during buzzing exceed those of the exposures mentioned above, failing to debilitate prey in the laboratory. Further, many toothed whales show evidence of chasing their prey over considerable distances (de Soto et al., 2008; Aoki et al., 2012); another observation that is inconsistent with the debilitation hypothesis. Thus, we conclude that whales do not debilitate prey with intense ultrasound, but use their high-amplitude clicks for locating and tracking their low target strength, mobile prey targets at long ranges.

## Defense strategies

To reduce or avoid predation by echolocating bats and toothed whales, prey can follow a variety of defense strategies (Brodie and Brodie, 1999). One of the primary defense mechanisms is to avoid being detected by the predator in the first place. In the case of an echolocating predator with acute hearing this can be achieved by acoustic crypsis, where the potential prey reduces the detection range of the echolocating toothed whale, either passively or actively.

The detection of the target prey can be impeded by a reduction in target strength or an increase in noise or clutter. Prey may thus reduce the detection range by minimizing the echo to noise/clutter ratio. Aquatic prey can accomplish this by seeking refuge among other echoic targets such as other organisms, the sea floor or rocks to hide acoustically between clutter or reverberation by which the echo of the prey is masked by other stronger echoes. This has also been seen in moths flying close to vegetation causing a reduction in the prey capture success of echolocating bats (Rydell, 1998). Prey can also have a small target strength and thereby decrease the echoes reflected back to the echolocating predator. Some toothed whales feed on deep water cephalopods, including members of *Histioteuthidae* and *Cranchiidae* (Clarke, 1996). These ammoniacal cephalopods have very little muscle mass and one of the consequences is a low target strength. They therefore produce a small echo compared to more muscular cephalopod species making them a more difficult target to detect (Madsen et al., 2007).

Some fish species are soniferous, which give the toothed whales the opportunity to eavesdrop on these sounds and use them as homing signals. Gulf toad fish have been shown to reduce or stop sound production when exposed to low-frequency dolphin sounds (Remage-Healey et al., 2006). This situation resembles that of potential bat prey using sound for their own intraspecific sexual communication, e.g., calling frogs (Tuttle and Ryan, 1981) or stridulating orthopterans (Belwood and Morris, 1987). Also here does the prey face the dilemma whether to keep on producing sounds to attract mates, at the risk of being eaten by the bat or to go silent at the risk of losing a mating (Belwood and Morris, 1987; Akre et al., 2011; Jones et al., 2011).

If a prey is detected, secondary defence mechanisms, such as startle behaviors and evasive manoeuvres function to reduce the risk of capture. In bat–insect interactions we find several examples of insects that are able to detect ultrasonic bat calls and exhibit evasive manoeuvres (Miller and Surlykke, 2001). Some moths from the family Arctiidae, tiger moths, have taken the defence strategies even further by emitting ultrasonic pulses when exposed to echolocation signals of bats. These anti-bat signals serve different purposes in different species of tiger moths; in some species they advertise moth toxicity, in others they startle the bat. It has recently been shown that anti-bat signals emitted by some tiger moths can also directly jam the bat biosonar (for a detailed review, see Conner and Corcoran, 2012). Similar examples of secondary defence strategies to toothed whale echolocation signals have not been found in marine prey species. The reason for this may be linked to the fact that secondary defence strategies require that the prey can detect the echolocation signals of the approaching predator; an ability that has evolved several times in insects, but seems to be quite rare in marine prey species as we shall see below.

## Ultrasound detection in marine prey

In contrast to overwhelming evidence of acoustic interactions between echolocating bats and their prey, our knowledge about toothed whales and their prey is sparse. Analysis of stomach contents show that toothed whales feed on a variety of different fish and cephalopod species (Simila et al., 1996; Santos et al., 2001a,b,c). However, only few studies have addressed if fish and cephalopods can detect the intense ultrasonic cues provided by echolocating toothed whales. Longfin squid (*Loligo pealeii*) do not show any detectable behavioral or neurophysiological responses when exposed to very intense ultrasound (Wilson et al., 2007; Mooney et al., 2010) and most fish species studied so far can only detect sounds up to some 500 Hz (Hawkins, 1981). Some fish species have specialized gas-filled structures in mechanical connection with their inner ears. These structures improve hearing sensitivity and extend the functional bandwidth up to frequencies between 3 and 5 kHz given by the resonance frequency of the gas-filled structures (Hawkins, 1981; Popper et al., 2003).

Despite this, recent experiments have shown that a few fish species can detect frequencies significantly higher than the resonance frequency of their swim bladder or other gas-filled structures in connection with their inner ears. Astrup and Møhl (1993) showed that conditioned cod would exhibit bradycardia when exposed to long ultrasonic pulses of 38 kHz above 203 dB re 1 μPa (pp). The authors suggested that these conditioned cardiac responses to ultrasound serve as evidence that cod can detect ultrasonic clicks emitted by echolocating toothed whales and might use the ability to reduce the risk of predation (Astrup and Møhl, 1993; Astrup, 1999). However, Schack et al. (2008) shed serious doubt on the findings of Astrup and Møhl (1993) by demonstrating that unconditioned cod do not exhibit any behavioral or cardiac responses when exposed to intense ultrasound. Schack et al. (2008) suggested that cod in the study of Astrup and Møhl (1993) were conditioned to low frequency or electrical artifacts rather than to the ultrasonic component of the exposure, and concluded that cod under natural conditions either fail to detect ultrasound or do not connote it with predation risk from toothed whales. Neither scenario would result in any reduction in the predation risks from ultrasound emitting toothed whales.

**Figure 2:**
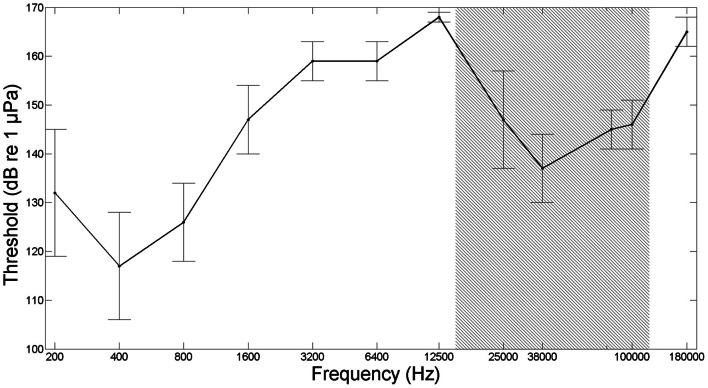
**Audiogram from American shad (*Alosa sapidissima*), based on conditioned cardiac responses in five fish.** Gray shaded area marks the frequency range of toothed whale echolocation signals (modified from Mann et al., 1997, 1998).

There are only few other studies reporting ultrasound detection in fish, and they are all based on Clupeiform fish species belonging to the subfamily Alosinae (Popper et al., 2004; Wilson et al., 2008). Kynard and O'Leary (1990) discovered that high frequency sonar at 160 kHz caused behavioral responses in migrating American shad (*Alosa sapidissima*). Subsequent studies, conducted in the search for an efficient way of keeping fish away from power plant water intakes, found that high frequency sounds at 110–160 kHz (180 dB re 1 μPa) were very effective in deterring Blueback herring (*Alosa aestivalis*) (Nestler et al., 1992) and alewives (*Alosa pseudoharengus*) (Dunning et al., 1992). However, it was debated if the fish actually detected the ultrasound or whether they detected low frequency byproducts of ultrasound emission. A few years later Mann et al. (1997, 1998) measured the first audiogram of an Alosinae, the American shad and showed that this species could detect ultrasound up to 180 kHz with a best sensitivity in the ultrasonic frequency range at around a frequency of 38 kHz and with a threshold of 146 dB re 1 μPa (pp) (Figure 2). Subsequent studies showed that other species belonging to the Alosinae, including the Gulf menhaden (*Brevoortia patronus*), allis shad (*Alosa alosa*) and twaite shad (*Alosa fallax*), can detect ultrasound (Mann et al., 2001; Gregory et al., 2007; Wilson et al., 2008, 2011). A few other Clupeiform fishes not belonging to the Alosinae have been tested for ultrasound detection, but with a negative outcome (Mann et al., 2001, 2005). From our current knowledge, the ability to detect ultrasound thus seems to be limited to only the 16 species of the subfamily of Alosinae, out of a total of more than 30,000 species of fish. Future studies will hopefully test for this by providing audiograms for more fish orders.

## Evasive reactions of alosinae

**Figure 3:**
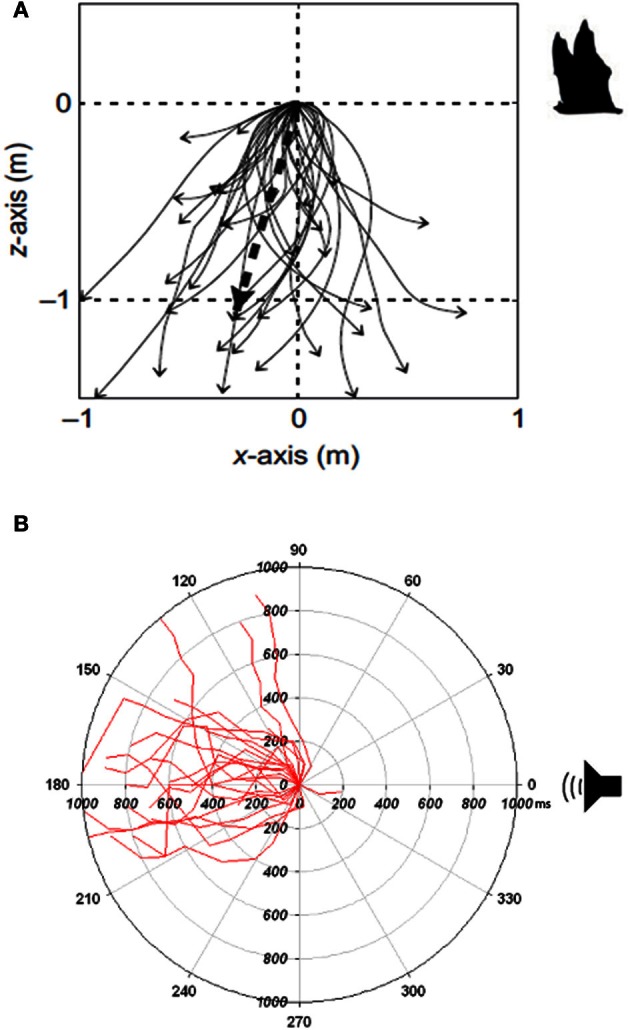
**(A)** Evasive dive profile of Grote's tiger moths (*Bertholdia trigona*) exposed to echolocating bats in a natural setting. The start position of the tiger moth is at 0,0 m when it starts a dive toward the ground (Adapted with permission from Corcoran and Conner, 2012). **(B)** Directional evasive maneuvers from allis shads (*Alosa alosa*) exposed to intense ultrasonic clicks played at a repetition rate of 250 clicks per second, mimicking echolocation signals from an approaching toothed whale in the buzz phase. The plot gives the angle between the sound source and the fish. At 0° the shad are facing the sound source (Adapted from Wilson et al., 2011).

Nestler et al. (1992) and Mann et al. (1998) speculated with inspiration drawn from studies on the acoustic interaction in air between bats and their prey (Roeder, 1962, 1967) that ultrasound detection in Alosinae serves as a defense against echolocating toothed whales. Behavioral studies conducted on American shad and allis shad in test tanks support this hypothesis: When shad are exposed to ultrasonic signals in the forms of either ultrasonic tones (Plachta and Popper, 2003; Wilson et al., 2008) or ultrasonic clicks mimicking the echolocation signals emitted by toothed whales (Wilson et al., 2011), they exhibit an escape response that is highly correlated with the intensity of the emitted signals. Wilson et al. (2011) exposed allis shad to ultrasonic click trains played with constant sound pressure levels, but with varying energy levels per time unit, generated by different repetition rates thereby mimicking a toothed whale at different phases of biosonar-based approach and capture. By keeping the sound pressure level constant and changing the click repetition rate, it was shown that the ultrasound detector in allis shad operates as an energy detector with a response threshold of 151 ± 6dB re 1 μPa^2^s. Furthermore when shad were exposed to ultrasonic click trains with high repetition rates, mimicking the buzz phase of a prey capture attempt of a toothed whale, the fish would exhibit a very strong response with high swimming speeds and faster reaction times. In contrast, when the repetition rate was decreased, mimicking a toothed whale at longer ranges, the response would be weaker and slower. The shad would, independent of the repetition rate and pre-exposure orientation, almost always turn away from the directional sound source at an angle of 180° (Figure 3). This behavior not only increases the distance to the toothed whale, but also make the shad a more difficult target to detect with sonar, as the target strength of a fish from the tail aspect can be reduced by up to 14 dB compared to a broad side aspect (Au et al., 2007). Swimming directly away results in a reduction of the detection range by 50% for the toothed whale (following Equation 1) (Wilson et al., 2011).

The response thresholds in allis shad are high, just like the response threshold in ultrasound sensitive moths (Surlykke et al., 1999). Such high response thresholds may reflect a trade-off between being caught by the predator, and the costs associated with unnecessary, but costly escape maneuvers. Despite the high detection threshold in ultrasound sensitive moths, Surlykke et al. (1999) estimated that a moth would be able to detect a bat at a range 10 times the range over which a bat would be able to detect the moth. A similar calculation for shad and a dolphin show that the shad would be able to detect a bottlenose dolphin at a distance of between 10–190 m (Mann et al., 1998; Wilson et al., 2011). In contrast to the moth–bat interaction, it is therefore likely that the bottlenose dolphin can detect a school of shad before the shad can detect the dolphin (Au et al., 2007). Still, the shad would be able to detect a bottlenose dolphin in the approach phase, well before it enters the final prey capture phase and most likely have sufficient time to take evasive actions.

Based on the behavioral experiments conducted in test tanks, the reaction of shad to ultrasound is consistent with it being an anti-predatory response against echolocating toothed whales. Like ultrasound sensitive insects, shads exhibit evasive maneuvers that are highly correlated in strength with the magnitude of the acoustically conveyed predation risk (Figures 3A,B).

## Ultrasound detectors

How Alosinae detect ultrasound has been an enigma since the ultrasonic sensitivity of these fish was discovered more than 15 years ago. Much more is known about the ultrasound sensitive ears of nocturnal insects that use mechanoreceptors as sound receivers in conjunction with tympanic membranes made of their cuticle at different positions on the body e.g., thorax (Notuidae), abdomen (e.g., Pyralidae) and mouthparts (e.g., Sphingidae) (Miller and Surlykke, 2001; Conner and Corcoran, 2012). In moths belonging to the family of Noctuidae, the anatomy of the ear is relatively simple: It consists of a thin tympanic membrane in a recess below the hind wing on the metathorax. A relatively large air sac, an expanded part of the respiratory system, is located behind the membrane. Mechanically coupled to the membrane are two mechanoreceptors, so called scolopidia, distinguished into A1 and A2. They attach to the same part of the tympanic membrane and are very similar in terms of their morphology and overall shape of their hearing threshold curves, but their sensitivity differ with A1 being approximately 20 dB more sensitive than A2 (Roeder, 1967; Fullard, 1998). When an ultrasonic sound wave impinges on the insect body, the membrane starts vibrating; this excites the sensory cells to increase their firing rate of action potentials (Roeder, 1967).

To evolve an ultrasonic pressure detector in water seems to be more challenging, perhaps because of the very different physical properties of air and water. Detection of ultrasound in water requires a gas-filled structure with wall properties that permit oscillations at ultrasonic frequencies. In addition, the gas-filled structure needs to be connected to a sensory receptor that can transduce the oscillatory motions into a neuro-electrical signal.

In all clupeiform fish, gas-filled structures (extensions from the swim bladder) are mechanically connected to two groups of mechanoreceptor hair cells, the lateral line and inner ear. The anterior part of the swim bladder has two gas-filled tubes that extend to the two inner ears, where they expand to gas-filled bullae encapsulated in bony structures (O'Connell, 1955; Retzius, 1881). The gas-filled bullae have a highly advanced structure (Wilson et al., 2009) and in most clupeiform fish, each bulla can be divided into a prootic bulla and a pterotic bulla (O'Connell, 1955). The function of the pterotic bulla is unknown, but the prootic bulla is believed to be an auditory specialization since it is connected to the utricle of the inner ear (O'Connell, 1955). The lateral line is also coupled to the prootic bulla. The perilymph of the prootic bulla and the sea water in the lateral line canals are only separated by the thin lateral recess membrane found in the back of the lateral recess, wherefrom the primary branches of the lateral line radiates (O'Connell, 1955; Denton and Blaxter, 1976; Hoss and Blaxter, 1982).

Enger (1967) suggested that the gas-filled bullae with mechanical connection to the utricle act as a pressure-to-displacement converter in Clupeidae. This makes Clupeidae sensitive to both the pressure and particle motion component of the sound field. The ability to detect the pressure component makes these fish capable of detecting higher frequencies and provides them with a more sensitive hearing (Hawkins, 1981). However, most clupeids can only detect sound below 10 kHz (Enger, 1967; Mann et al., 2001, 2005). Since the gas-filled bullae in addition are mechanically connected to the lateral line, it has been suggested that vibrations of the bullae also generate fluid motions in the cephalic lateral line canals, and thereby cause a deflection of the hair cells in the neuromasts of the lateral line (Denton and Blaxter, 1976; Denton and Gray, 1983; Gray, 1984).

The mechanical connections between the lateral line, the inner ear and the gas-filled bullae in clupeids are unique. It is therefore tempting to hypothesize that the ultrasound detector in Alosinae is associated with the unique bullae complex, and that the gas-filled bullae are acting as a transducing element that translate the ultrasonic pressure wave into a local particle motion stimulating the sensory receptor (Higgs et al., 2004). The gas-filled bullae are indeed involved in ultrasound detection in the Alosinae: Wilson et al. (2009) showed that the gas-filled bullae in Gulf menhaden pulsate when placed in an ultrasonic sound field, and furthermore that replacement of gas in the bullae with fluid eliminates the ability to detect ultrasound. Since the bullae are connected to both the inner ear and the lateral line, it is possible that the sensory receptor is part of either the lateral line or the inner ear.

Mann et al. (1998) suggested that the utricle of the inner ear is where the ultrasound sensory receptor in Alosinae is located because of the highly advanced anatomy. Higgs et al. (2004) found morphological differences in the sensory epithelium of the utricle between Alosinae and other clupeids. The sensory epithelium of the utricle in Clupeidae is divided into three parts; the anterior, posterior and middle (Popper and Platt, 1979). However, the anatomical support for the middle section of the sensory epithelium in Alosinae is thinner and therefore more loosely connected to the rest of the maculae compared to other clupeids. Higgs et al. (2004) speculated that the looser connection could make the utricle sensitive to high frequency vibrations induced by the gas-filled prootic bullae and the elastic thread. Despite of this, there is no experimental evidence to support that the utricle mediates ultrasound detection.

**Figure 4:**
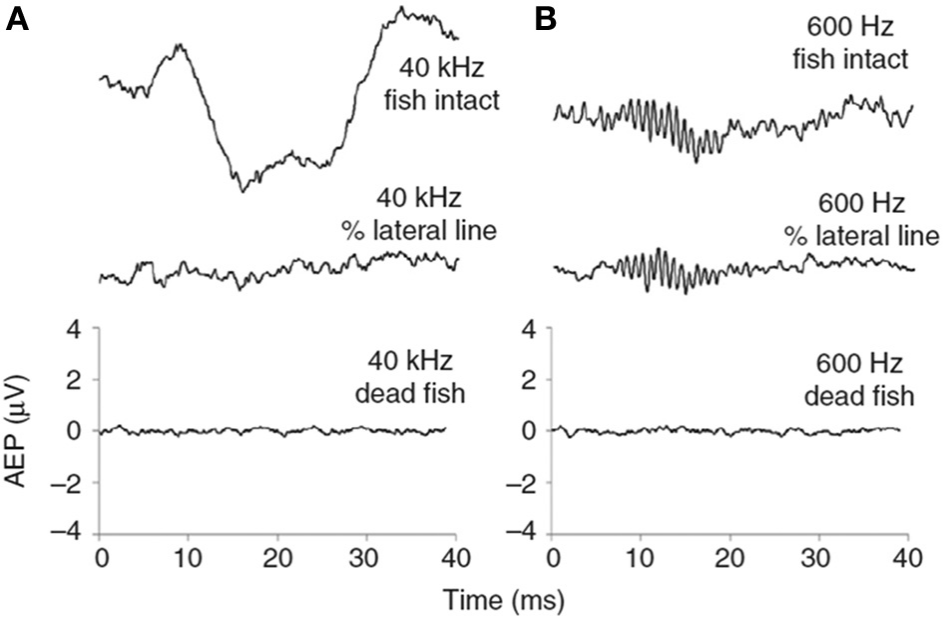
**Neural responses of a Gulf menhaden measures as evoked potentials to (A) a 40 kHz and (B) a 600 Hz 20 ms long tone before and after mechanical manipulation of the lateral line. Adapted from Wilson et al., 2009**.

Another theory suggests that the ultrasound sensory receptor is associated with the lateral line (Nestler et al., 1992; Wilson et al., 2009). This theory is supported by the observation that the neural response to ultrasound disappears by mechanical manipulation of part of the lateral line overlying the base of the lateral line, i.e., the lateral recess. This manipulation does not damage neither the gas-filled bullae nor the inner ear, as evidenced by the ability to detect a 600 Hz tone after this manipulation. Therefore the lateral line plays an important role in ultrasound detection and the most parsimonious explanation is that the sensory receptor is either to be found in the lateral line or in association with the lateral line (Wilson et al., 2009) (Figure 4).

## Why is ultrasound detection so rare in aquatic prey compared to in nocturnal insects?

In water ultrasound detectors have as far as we know only evolved in very few fish species, the Alosinae, whereas most fish and cephalopods, and most likely also crustaceans, cannot detect intense ultrasound. This is in contrast to the situation for insects in air, where ears sensitive to ultrasound have evolved in many orders of nocturnal insects independently (Miller and Surlykke, 2001; Conner and Corcoran, 2012). Despite a remarkable evolutionary convergence in the biosonar behavior and frequency range of echolocation signals from bats and toothed whales, the evolution of ultrasound detection in prey is much rarer in water than in air.

This may be due to the fact that the two groups of echolocating predators have evolved in two very different media and therefore the functional starting points for the evolution of ultrasound detection in their prey is very different. Ultrasound reception requires detection of the pressure component of a sound field. That in turn calls for receptors with structures having an impedance difference compared to the surrounding medium. In simple insect ears the large impedance difference between the surrounding air and the insect body in combination with the air sac behind the tympanic membrane, generates vibrations of the membrane relative to the rest of the insect body when insects are exposed to ultrasound (Roeder, 1967). In fact, non-differentiated mechanoreceptors attached to the cuticle are sensitive to airborne sound with a best frequency of around 2 kHz at sound pressures above ca. 80 dB SPL (Yack and Fullard, 1990). Thus, the precursor for an ear is readily available in insects. In water, the situation is quite different: A fish or cephalopod without gas-filled structures in the body is almost acoustically transparent, since their bodies have impedance close to the impedance of the surrounding water. Fish and cephalopod have evolved a low frequency hearing system, where deflections of hair cells are caused by differential motion of dense ear stones with respect to the hair cells and the rest of the body (Sand and Karlsen, 2000). However, this accelerometer ear is in most species sensitive only up to a few hundreds of Hz (Kalmijn, 1989). Several fish species have, in addition to the accelerometer ear, gas-filled structures, such as the swim bladder, mechanically connected to their inner ears. These gas-filled structures render the fish sensitive to the pressure component of the sound field and hence capable of detecting frequencies higher than the resonance frequency of their otolith organs. Still, even though some fish have a strong mechanical connections between their ears and the gas-filled structures, they can only hear up to 3–5 kHz (see review by Hawkins, 1981; Popper et al., 2003). The only exception found so far is the subfamily Alosinae that are capable of detecting intense ultrasound with their gas-filled bullae complex. Thus, evolving an ultrasound detector in fish and cephalopod seems to require challenging anatomically adaptations compared to insects, and this might be one of the reasons why ultrasound detection in marine species seems limited to Alosinae.

**Figure 5:**
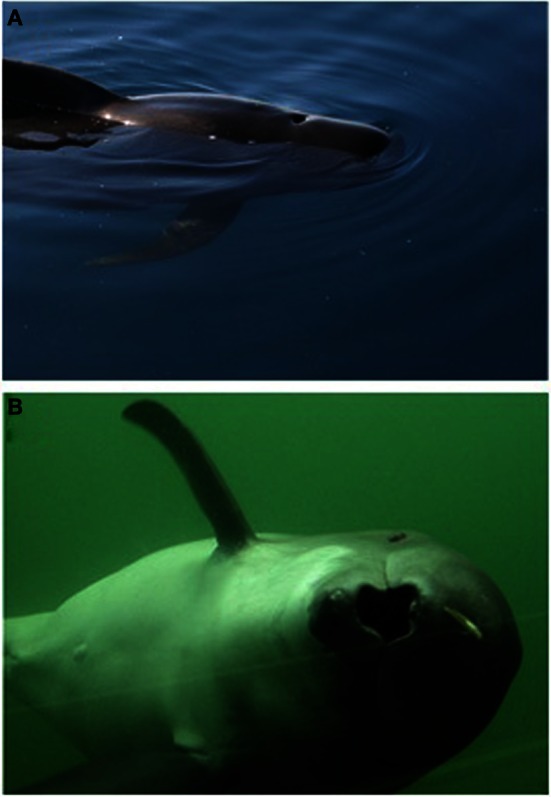
**(A)** A short-finned pilot whale (*Globicephala macrorhynchus*) swimming at the surface showing the hydrodynamic disturbances generated by the forward motion of the whale (photo: Frants H. Jensen). **(B)** A suction-feeding harbor porpoise (*Phocoena phocoena*) (photo: Fjord and Bælt, Kerteminde, Denmark).

Another and perhaps even more important difference might be found in different selection pressures working in the two acoustic interactions. The selection pressure for evolving ultrasound detectors is presumably very high for the nocturnal insects, since bats are the only nocturnal insectivores that hunt prey on the wing. In contrast, toothed whale prey is also targeted by a plethora of other marine predators that employ a range of sensory and locomotory means to subdue their prey. Therefore toothed whales are not an exclusive group of predators placing a one-sided evolutionary selection pressure to evolve means to detect and evade them. In addition to the ultrasonic echolocation signals, toothed whales also produce another acoustic cue that is shared with all other aquatic predators: Low frequency hydrodynamic water movements are consistently generated during aquatic feeding by both swimming motions, the head wake of the approaching predator and the subsequent suction and raptorial feeding motions during prey acquisition (Hanke and Bleckmann, 2004; Fish and Lauder, 2006; Werth, 2006) (Figure 5). Therefore toothed whales and other aquatic predators provide the prey with strong low frequency cues and the selection pressure for evolving means to detect such cues will presumably be much stronger than the selection pressure to evolve means to detect the ultrasonic cues, because of the universality of this low frequency cue (Vermeij, 2002). The ability to detect infrasonic cues is likely found in most water living metazoans, including copepods (Heuch and Karlsen, 1997), cephalopods (Packard et al., 1990) and bony fish (Sand and Karlsen, 1986; Karlsen, 1992a,b; Karlsen et al., 2004). One of the major driving forces for evolving an acute infrasonic hearing sensitivity might be the necessity for detection of predators (Sand and Karlsen, 2000). Playback studies testing behavioral escape responses of fish when exposed to infrasound mimicking an approaching predator supports this hypothesis, since different fish species exhibit a strong spontaneous avoidance response when they are exposed to infrasound with no or little sign of habituation (Knudsen et al., 1994; Sand et al., 2001; Karlsen et al., 2004).

## Conclusion

Despite the very different physical environments, the evolution of echolocation in bats and toothed whales seem surprisingly convergent in terms of spectral and temporal acoustic features of the sonar signals: Toothed whales and bats operate their biosonar in the same frequency range and with overlapping sample rates. In both scenarios a prey capture involves characteristic changes in particular of time but also intensity parameters to subdivide the pursuit into three phases; search, approach, and finally the buzz phase with extremely high pulse rate just before the prey is caught. The acoustic interaction between bats and their prey, the nocturnal insects, has become a textbook example of an evolutionary arms race between a predator and its prey. Currently available data does not indicate that a similar ultrasonic interaction exists between toothed whales and the majority of their prey.

Predation defense in terms of ultrasonic detection of echolocators seem far from as common among fish and cephalopod prey of toothed whale compared to the insect prey of bats. Still, there are some clear similarities in the anti-predator responses of one group of prey fish, the Alosinae (shad and menhaden), to those of eared nocturnal insects like e.g., moths. The strength of the evasive maneuvers is highly correlated with the magnitude of the acoustical signals conveying a predation risk. If a moth or a shad is exposed to weak echolocation signals mimicking a bat or a toothed whale at a distance, the evasive maneuver consists of a directional response away from the source. However, if the sound exposure is mimicking a bat or a toothed whale nearby, the evasive maneuver is stronger and unpredictable. The behavioral response thresholds for both moths and Alosinae are relatively high, but possibly low enough to provide enough time to successfully escape the predator, while high enough to reduce the number of false, and hence expensive, alarms.

While the ability to detect ultrasound has evolved in many insect families, it has so far only been described in a few fish species belonging to the subfamily Alosinae. In the bat–insect interaction the selection pressure among insects for evolving means to detect and avoid the bat is high, because essentially all nocturnal predation on flying insects stems from these predators. In the interaction between toothed whales and their prey the selection pressure seems much weaker, most likely because toothed whales are by no means the only marine predators placing a selection pressure on their prey to evolve specific means to detect and avoid them. Toothed whales, like all other aquatic predators, produce an omnipresent low frequency sensory cue that can be detected by fish and cephalopods. The selection pressure is presumably stronger to evolve means to detect the low frequency cues, than to develop ultrasound detectors. This is supported by the fact that in all fish and cephalopod species studied up to date we find a high sensitivity to low frequency particle acceleration that may represent an interface for an acoustic arms race between not only toothed whales, but all aquatic predators and their prey.

### Conflict of interest statement

The authors declare that the research was conducted in the absence of any commercial or financial relationships that could be construed as a potential conflict of interest.
